# ADHD detection from EEG signals using GCN based on multi-domain features

**DOI:** 10.3389/fnins.2025.1561994

**Published:** 2025-04-04

**Authors:** Ling Li, Xueyang Guo, Zihan Yang, Yanping Zhao, Xu Liu, Junxian Yang, Yanyan Chen, Xinxian Peng, Lina Han

**Affiliations:** ^1^College of Communication Engineering, Jilin University, Changchun, China; ^2^College of Geo-exploration Science and Technology of Jilin University, Changchun, China; ^3^Changchun Sixth Hospital of China, Changchun, China

**Keywords:** ADHD, EEG, brain functional connectivity, multi-domain features, GCN

## Abstract

**Introduction:**

Attention deficit hyperactivity disorder (ADHD) is a common psychiatric disorder in children during their early school years. While many researchers have explored automated ADHD detection methods, developing accurate, rapid, and reliable approaches remains challenging.

**Methods:**

This study proposes a graph convolutional neural network (GCN)-based ADHD detection framework utilizing multi-domain electroencephalogram (EEG) features. First, time-domain and frequency-domain features are extracted via long short-term memory (LSTM) and convolutional neural network (CNN) models, respectively. Second, a novel functional connectivity matrix is constructed by fusing phase lag index (PLI) and coherence (COH) features to simultaneously capture phase synchrony and signal intensity consistency between brain regions. Finally, a GCN model integrates these time-frequency features with topological patterns from the connectivity matrix for ADHD classification.

**Results:**

Evaluated on two EEG datasets, the proposed method achieved average accuracies of 97.29% and 96.67%, outperforming comparative models (XGBoost, LightGBM, AdaBoost, random forest). Visualization experiments further revealed distinct brain connectivity distributions between ADHD patients and healthy controls.

**Discussion:**

The fused functional connectivity matrix surpasses traditional single-metric approaches in characterizing brain interactions. By synergistically combining time, frequency, and topological features, the GCN framework enables more precise ADHD detection. This method demonstrates potential for assisting neurologists in clinical diagnosis while providing interpretable neurophysiological insights.

## 1 Introduction

Attention deficit hyperactivity disorder (ADHD) is a neuropsychological and behavioral disorder that commonly develops in children during their early school years and has been listed as an important category of children’s developmental behavior problems (Leffa et al., 2022). According to epidemiological data, about 5–7% of children worldwide are affected by ADHD. Children with ADHD often have difficulty concentrating, establishing friendships with peers, and have high mood swings, which can have a great negative impact on children’s lives (Konrad et al., 2010). Accurate and rapid diagnosis of ADHD will be beneficial for clinical research to explore more effective treatment methods. Over the years, researchers have used various neuroimaging modalities and analytical methods to gain deeper information about ADHD (Samea et al., 2019). Among them, electroencephalography (EEG) has become the main means of ADHD research due to its advantages of high temporal resolution, portable equipment, and low cost. As a comprehensive reflection of the electrophysiological activities of brain nerve cells, EEG signals present rich information on the cerebral cortex or scalp surface (Evans-Lacko et al., 2018). At present, many medical institutions have taken the abnormal performance of EEG as an important indicator for the auxiliary diagnosis of ADHD.

With the rapid development of machine learning technology, the field of medical diagnosis has ushered in a new revolution (Bhavsar et al., 2021). Especially in the detection of mental diseases, the combination of EEG-based analysis methods and machine learning technology has become a research frontier and hot spot, and this fusion technology is expected to provide strong support for the early diagnosis and precise treatment of diseases such as ADHD (Cura et al., 2024). Trinh et al. (2023) used closed-eye resting EEG data to extract the relative spectral power of 12 frequency bands from 64 EEG channels and four machine learning models including support vector machine, k-nearest neighbor, random forest and elastic network, to compare their detection performance for ADHD. Ahire et al. extracted the morphological features and power spectral density features using the resting state EEG data with eyes open EEG data, and used principal component analysis (PCA) to reduce the data dimension. Finally, machine learning classifiers such as Adaboost, k-nearest neighbor, naive bayes and random forest were utilized to classify ADHD patients (Ahire et al., 2023). Maniruzzaman et al. (2023) proposed a hybrid channel selection method and used a Lasso logistic regression-based model to select important features from the selected channels, and finally applied multiple machine learning classifiers including Gaussian process classification (GPC), multilayer perceptron, decision trees, logistic regression and other machine learning classifiers to identify children with ADHD.

Recently, more and more deep learning models are widely used in the ADHD classification (Mao et al., 2019). Deep learning methods can automatically extract the features that include almost all the information of the data, thus avoiding the tedious feature screening process. In contrast, traditional machine learning algorithms often have differences in how they handle features, resulting in a lack of necessary links between findings (Cicek and Akan, 2021). Zhang et al. (2022) proposed a convolutional neural network-long short term memory (CNN-LSTM) model, which included temporal convolution module, spatial convolution module and long short-term memory module, and achieved good classification results in the classification tasks of ADHD and healthy controls. Alkahtani et al. (2023) extracted the time, frequency, information theory and other features of each electrode in each frequency band of EEG signals, used recursive feature elimination (RFE) and Lasso regularization methods for feature selection, and selected multi-layer perceptron (MLP) model and convolutional neural network (CNN) model for training and test. Jahani and Safaei (2024) firstly extracted features from EEG signals, then trained them with CNN and residual neural network, and found that residual neural network had better classification result.

Furthermore, the research methods for EEG medical diagnosis have gradually changed from focusing on the capture of single channel features to exploring multi-channel connection patterns (Chen et al., 2019). A growing body of research suggests that brain connectivity can reveal the functions and the complex cortical communication of different brain regions, which may lead to better research on many psychiatric disorders such as ADHD (Allen et al., 2014; Babiloni et al., 2005; Drysdale et al., 2017; Fu et al., 2021). In recent years, a number of studies have attempted to address the question of brain connectivity. Bakhtyari and Mirzaei (2022) proposed a new feature extraction scheme based on the evaluation of the dynamic connection tensors between EEG channels, and used a neural network model composed of the long and short term memory (LSTM) network and attention mechanisms for ADHD detection. Polat (2023) used the phase lag index (PLI) to construct a brain functional connectivity matrix and used residual neural networks to achieve automatic diagnosis of schizophrenia. Li et al. (2024) fused coherent values and phase locking values (PLV) of synchronous compressed wavelet as new markers to build a functional connectivity matrix, and used CNN to achieve accurate identification of major depressive disorder. Xu et al. (2024) used the Pearson correlation coefficient (PCC) to quantify functional connectivity among EEG channels, mapped the expanded functional connectivity matrix into a time series graph, and finally used CNN-LSTM to recognize autism spectrum disorders.

However, the research based on the brain functional connectivity of multi-channel EEG signals has still few applications for ADHD detection. Moreover, it is well known that the method of constructing the functional connectivity matrix using phase synchronization only depends on the phase difference between the signals and does not take into account the amplitude, so the effect of the change in signal strength may be ignored. To address this issue, we propose an ADHD detection method using a graph convolutional neural network (GCN) model based on multi-domain features from multi-channel EEG signals in this paper. In this method, we firstly extract time domain and frequency domain features by the LSTM and CNN models respectively, and then develop a fusion feature based on the PLI and coherence (COH) that can obtain both phase synchronization and amplitude coherence and use it as a new index to construct the functional connectivity matrix of brain network. Subsequently, in order to integrate more useful EEG information, a GCN model combining the features extracted by the LSTM and CNN models and the functional connectivity features is designed as the classification model, which not only takes into account both time and frequency information, but also has strong topological feature extraction ability, resulting in improving the accuracy and reliability of ADHD detection. The main contributions of this study are summarized as follows:

1. The features in time and frequency domains are extracted using the LSTM and CNN models, respectively, from multi-channel EEG signals.
2. The functional connectivity matrix representing brain functional connectivity is constructed by fusing the PLI and COH features, which can simultaneously reflects the phase synchronization and consistency of signal intensity between different brain regions.
3. A GCN classification model combining the LSTM and CNN models and brain functional connectivity is developed for ADHD detection, which improves the performance of proposed detection method by extracting time domain, frequency domain and topological features.
4. Finally, performance comparisons of the proposed method and other methods are performed on our dataset and a public dataset, respectively.

The rest of this article is organized as follows. The “Materials and methods” section includes the descriptions of datasets, feature extraction, classification model and performance metrics. The “Experimental results and discussion” section focuses on comparing results, visualizing brain connectivity patterns, and the limitations and future development of current research. The “Conclusion” section discusses the feasibility of the proposed method and summarizes the research.

## 2 Materials and methods

**Figure 1:**
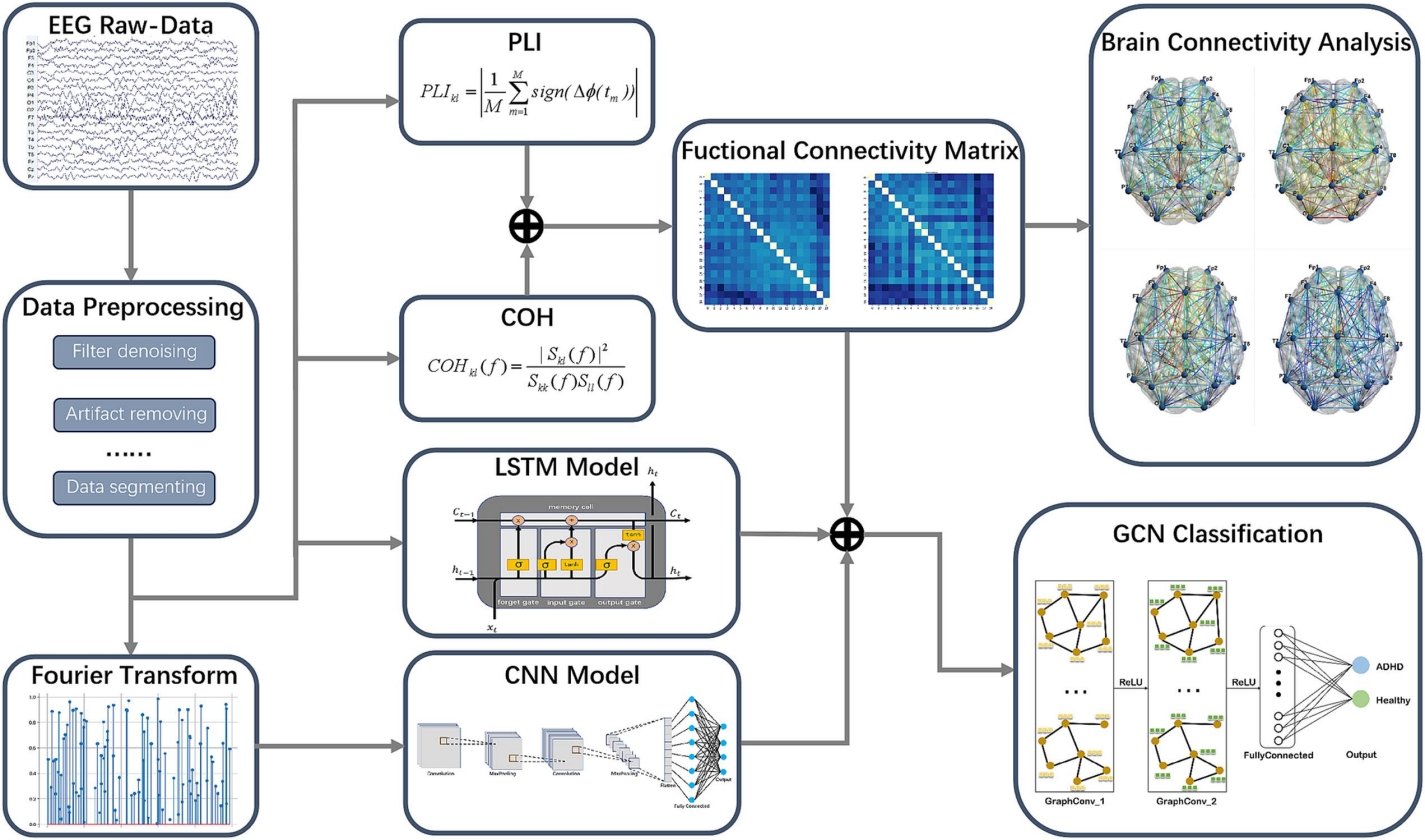
The implementation process of the proposed ADHD detection method.

EEG signals contain a wealth of information, in the medical field we can make full use of EEG signals to identify mental diseases. In this study, we design a GCN framework based on EEG by combining multi-domain features to detect ADHD. The implementation process of the proposed ADHD detection method is shown in Figure 1. First we preprocess the EEG data, and use the LSTM model to extract the time domain features. Then we perform Fourier transform on the processed time-domain data to obtain the frequency-domain data, after which we input them into the CNN model to extract the frequency domain features. Next, we combine the PLI and COH as a fusion feature to construct a new functional connectivity matrix that is the brain functional connectivity features. Finally, we input the above three kinds of features together into the GCN model for training and classification. In addition, we conduct visualization experiments on brain connectivity to explore the differences of brain connectivity patterns in ADHD.

### 2.1 Datasets and pre-processing

To better verify the validity of the proposed approach, we use two completely different datasets. One is our own collection, the other is a public dataset. The detailed descriptions are as follows.

Dataset 1 was collected from January 2023 to January 2024 at the Changchun Sixth Hospital of China. A total of 57 participants were recruited, including 29 children with ADHD (17 boys and 12 girls; Ages 4–13) and 28 healthy children (20 boys and 8 girls; Ages 4–13). ADHD children were diagnosed by an experienced child and adolescent psychiatrist according to the Diagnostic and Statistical Manual of Mental Disorders, Fourth Edition (DSM-IV) criteria (Cooper, 2001). The clinical diagnosis based on the DSM-IV was recorded as the DSM score, which is the mean of the total scores of all the indicators. It is greater than one that indicates the presence of ADHD and the higher score the more severe the ADHD, while the score is less than one that indicates the absence of ADHD. Details of the healthy control and ADHD groups in Dataset 1 are shown in Table 1. The healthy children in the control group underwent rigorous physical health screening and confirmed that they had not taken any drugs during the test period, and had no mental abnormalities, learning and growth problems. The participants were instructed not to drink coffee or abuse other drugs. The parents introduced the experiment to the test children and put them in a relaxed state. Nicolet’s 19-lead 10–20 EEG acquisition device was used to capture at least five minutes of data with their eyes closed. The sampling frequency was set at 580 Hz and the electrode impedance was controlled below 80 kΩ. The 19 electrodes covered the scalp with the international 10–20 electrode placement standard (Klem et al., 1999), including the frontal lobe (Fp1, Fp2, F3, F4, F7, F8, Fz), temporal lobe (T7, T8), parietal lobe (P3, P4, P7, P8, Pz), occipital lobe (O1, O2), and central (C3, C4, Cz) regions. So 19 channels EEG signals were recorded.

**Table 1:** Details of the healthy control and ADHD groups of Dataset 1.

Characteristic	Control	ADHD
Male [nos.]	20	17
Female [nos.]	8	12
Age [years]	9.44 (±1.70)	9.65 (±1.97)
DSM score	0.24 (±0.36)	1.53 (±0.67)

Dataset 2 is a publicly available dataset (Nasrabadi et al., 2020). The dataset was collected including 61 children with ADHD (48 boys and 13 girls; Aged 7–12) and 60 healthy children (50 boys and 10 girls; Ages 7–12). ADHD children were also diagnosed by an experienced psychiatrist according to DSM-IV criteria and had been treated with Ritalin for up to 6 months. Children in the control group did not have any history of psychiatric disorders, epilepsy, or high-risk behaviors. Due to visual attention deficits in ADHD children, EEG recording protocols revolved around visual attention tasks. In the task, children were shown a set of cartoon characters and asked to count the number of characters. The number of people in each picture was randomly between 5 and 16, and the images were large enough for children to easily identify and count. To maintain continuity during signal recording, each picture was displayed without interruption immediately after the child responded. Thus, the duration of the EEG recording throughout the cognitive-visual task depended on the child’s performance (i.e., response speed). EEG recording also followed a 10–20 system and was performed at a sampling frequency of 128 Hz through 19 channels including Fz, Cz, Pz, C3, T3, C4, T4, Fp1, Fp2, F3, F4, F7, F8, P3, P4, T5, T6, O1, and O2. The names of the four electrodes in the two datasets are different, but their corresponding electrode positions are the same: T7, P7, P8, T8 (Dataset 1) corresponds to T3, T5, T6, T4 (Dataset 2).

We used the EEGLAB toolbox for data preprocessing. First, the EEG data were calibrated with electrode positions. Subsequently, the EEG signals were filtered using a bandpass filter from 0.5 Hz to 60 Hz, and the sampling frequency was adjusted to 128 Hz. To further remove artifacts, independent component analysis (ICA) and ICLable algorithm were used to identify artifacts. Finally, we split the raw EEG signal into non-overlapping 5-s segments for expanding the available data, resulting in 3477 and 3,322 segments in Dataset 1 and Dataset 2, respectively.

### 2.2 Feature extraction

#### 2.2.1 Time domain feature

In this paper, we use a model composed of the LSTM network to extract time domain features of multi-channel EEG signals. LSTM network is a special type of recurrent neural network (RNN), which can effectively capture long-term dependence in sequence data by introducing gating mechanism, and overcome the problem of gradient disappearance for processing long sequences in traditional RNN. The network structure is particularly suitable for processing time series data and can capture complex timing patterns.

**Figure 2:**
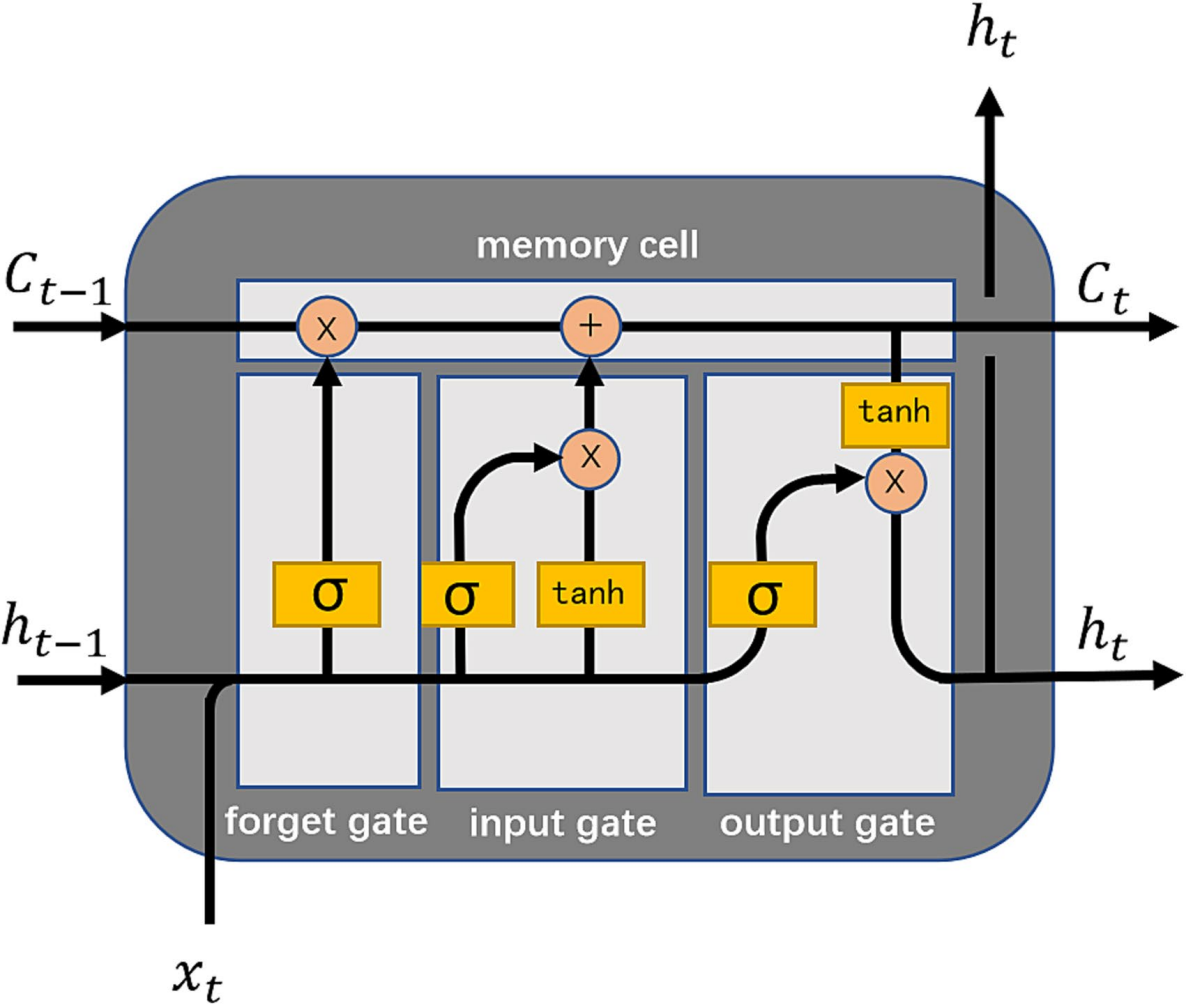
The LSTM network schematic.

The structure of LSTM network consists of a forget gate, an input gate, a memory cell and an output gate. The memory cell is the “memory” of the LSTM network, the forget gate controls the retention of old information, the input gate controls the addition of new inputs, and the output gate determines what information will be passed on the next time. Figure 2 shows the LSTM network schematic.

Specifically, the LSTM network realization process is as follows.

##### 2.2.1.1 Forget gate

The purpose of the forget gate is to decide what information should be forgotten or retained from the memory cell at each time. It is calculated by


(1) $$f_{t}=\sigma \left(W_{f}\left[h_{t-1},x_{t}\right]+b_{f}\right)$$

where $f_{t}$ (Equation 1) is the output of the forget gate at current time t , sigmoid function $\sigma \left(\cdot \right)$ is the activation function, which compresses the input values between 0 and 1, $\left[\cdot \right]$ means to splice two vectors, $W_{f}$ , $h_{t-1}$ , $x_{t}$ and $b_{f}$ are the weight matrix, the hidden state at the previous time t−1 , the input at current time t and bias term, respectively.

##### 2.2.1.2 Input gate

The input gate consists of two parts, a sigmoid layer that decides which values will be updated, and a tanh layer that creates a new candidate value vector that will be added to the memory cell state. The formula for the input gate is


(2) $$i_{t}=\sigma \left(W_{i}\left[h_{t-1},x_{t}\right]+b_{i}\right)$$

(3) $${\tilde{C}}_{t}=\tanh\left(W_{C}\left[h_{t-1},x_{t}\right]+b_{C}\right)$$

where $i_{t}$ (Equation 2) is the output of the input gate, ${\tilde{C}}_{t}$ (Equation 3) is the candidate memory cell state, Hyperbolic tangent function $\tanh\left(\cdot \right)$ compresses the input value between −1 and 1, and $W_{i}$ , $W_{C}$ , $b_{i}$ , $b_{C}$ are the relevant weights and biases, respectively.

##### 2.2.1.3 Memory cell

The memory cell is the core of the LSTM network and is responsible for maintaining and updating long-term dependent information during the whole sequence processing process. The memory cell state of the current time is determined by the memory cell state of the previous time and the candidate memory cell state. The memory cell state is


(4) $$C_{t}=f_{t}⊙C_{t-1}+i_{t}⊙{\tilde{C}}_{t}$$

where $C_{t}$ (Equation 4) is the memory cell state of the current time t , $C_{t-1}$ is the memory cell state of the previous time t−1 , and ⊙ represents Hadamard product, that is the product of corresponding elements in two vectors.

##### 2.2.1.4 Output gate

The output gate is responsible for determining which part of the memory cell state will be output to the hidden state, which is calculated by


(5) $$o_{t}=\sigma \left(W_{o}\left[h_{t-1},x_{t}\right]+b_{o}\right)$$

(6) $$h_{t}=o_{t}⊙\tanh\left(C_{t}\right)$$

where $o_{t}$ (Equation 5) is the output of the output gate, $h_{t}$ (Equation 6) is the hidden state at the current time t , $W_{o}$ and $b_{o}$ are the weight and bias of the output gate, respectively.

We use a LSTM model to extract time domain features by adding a fully connected layer after a LSTM layer. To avoid overfitting, each layer uses dropout with certain probability. Table 2 shows the parameter descriptions for the LSTM model.

**Table 2:** Parameter descriptions for the LSTM model.

Layer-model	LSTM
Long short-term memory	128
Dropout	50%
Fully-connected	64
Dropout	50%
Output	2

In this paper, the input $x_{t}$ of the LSTM model is the 5-s segment of EEG signal after preprocessing, and the output is a two-dimensional time-domain feature vector for every channel. For 19 channel EEG signals, we obtain a 19×2 feature matrix. The LSTM model is trained by a binary cross-entropy loss function using an Adam optimizer with 10 epochs and a batch size of 32.

#### 2.2.2 Frequency domain feature

**Figure 3:**
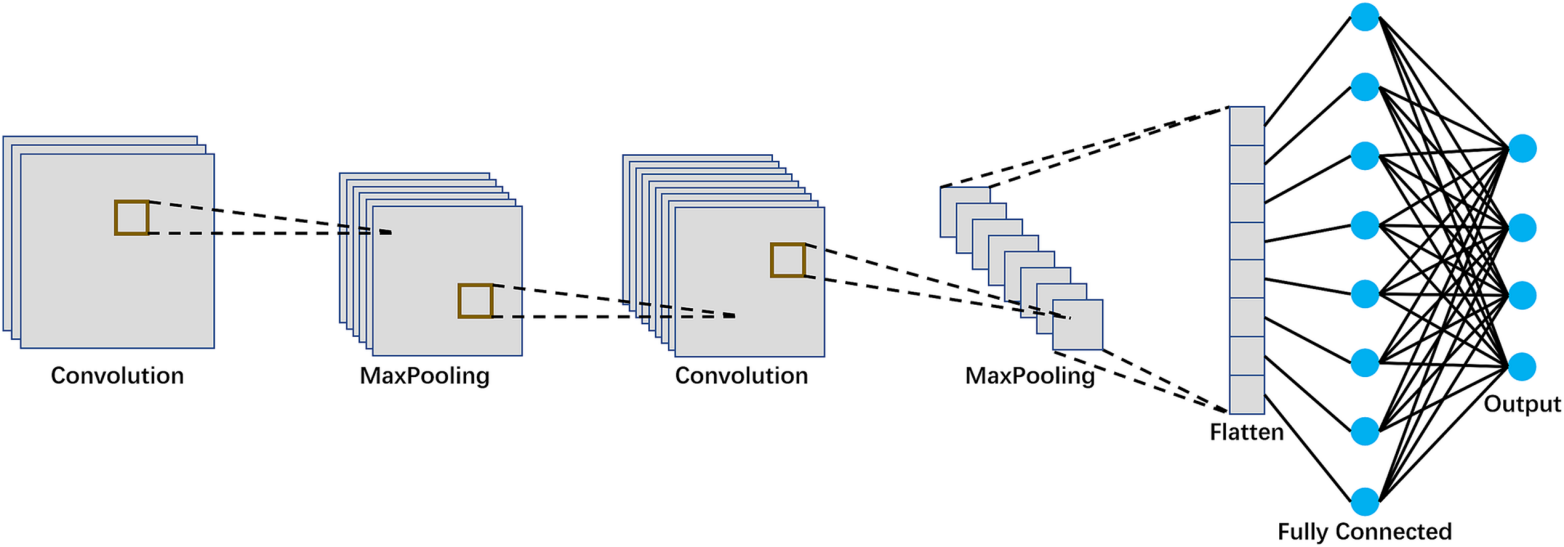
The flowchart of the CNN model.

In order to extract frequency domain features, we convert EEG signal into the frequency domain by the Fourier transform (FT) for each EEG channel, and then obtain the spectrum graph. Then we use CNN to extract frequency features. Figure 3 shows the flowchart of the CNN model.

The realization process of the CNN model is as follows:

##### 2.2.2.1 Convolution layer

This layer is responsible for extracting local frequency features. On the convolution layer, multiple convolution kernels are convolved with input data, and a series of feature graphs can be obtained through an activation function after biasing (Bouvrie, 2006).

##### 2.2.2.2 Pooling layer

Like convolutional layers, pooling layer plays an important role in the CNN model. This layer is often connected behind the convolution layer, and the features are downsampled to reduce the dimension of the feature graph. Pooling layer generally only performs dimensionality reduction operation without parameters updating. On this layer, the output of the convolution layer, that is, feature graph, which is pooled in each n×n region with a non-overlapping size. Then the maximum value or average value of each region is selected, and finally the output feature is reduced by n times in both dimensions.

##### 2.2.2.3 Fully connected layer

After the input data is alternately propagated through multiple convolutional layers and pooling layers, the extracted features are outputted by a fully connected layer. On the fully connected layer, the vector obtained by flattening the feature graph after pooling layer is weighted and summated by an activation function.

CNN can effectively capture the features in the frequency domain by sensing the local spectrum graph. In this paper, a CNN model is used to extract the frequency features, which includes two convolution layers, two pooling layers and a fully-connected layer. Dropout is used after the convolution layer and fully-connected layer for avoiding overfitting. Table 3 shows the parameter descriptions for the CNN model.

**Table 3:** Parameter descriptions for the CNN model.

Layer-model	CNN
Convolution	16–1 * 1 strides
Dropout	50%
Pooling	2–2 * 2 strides
Convolution	32–1 * 1 strides
Dropout	50%
Pooling	2–2 * 2 strides
Fully-connected	64
Dropout	50%
Output	2

In this paper, the number of points of the FT is 640, the size of convolution kernel is 5×5 , maxpooling is used, n=2 , the activation function is ReLU . The CNN model is trained by a binary cross-entropy loss function using an Adam optimizer and the number of epochs is set to 10 and batch size is 32. The input of the CNN model is a 19×640 spectrum matrix, and the output is a 19×2 frequency-domain feature matrix for 19 channels.

#### 2.2.3 Functional connectivity

##### 2.2.3.1 Phase lag index

The phase lag index (PLI) is a measure of phase synchronization between two signals, which is particularly suitable for detecting true phase synchronization in EEG analysis without volume conduction effects. The volume conduction effect is a common problem in EEG signal processing because it can cause signals measured from different electrodes to appear pseudo-synchronized (Clemens et al., 2016). The PLI avoids this pseudo-synchronous interference by focusing on situations where the phase difference between the two signals is not equal to 0 or π . The PLI (Equation 7) of signals between the kth channel and the lth channel is defined as


(7) $$\mathrm{PL}I_{\mathrm{kl}}=|\frac{1}{M}\overset{M}{\underset{m=1}{\sum }}\mathrm{sign}\left(\Delta \phi \left(t_{m}\right)\right)|$$

where $\mathrm{sign}\left(\cdot \right)$ denotes symbolic function, $\Delta \phi \left(\cdot \right)$ represents the phase difference of the two channel signals $x_{k}\left(t\right)$ and $x_{l}\left(t\right)$ , 1≤k,l≤K . K is the number of channels, and M is the number of the sample points of signals $x_{k}\left(t\right)$ and $x_{l}\left(t\right)$ .

The PLI is one of the important brain functional connectivity indicators. The range of PLI values is from 0 to 1 with larger values indicating stronger phase synchronization between signals. For K channel EEG signals, a K×K PLI functional connectivity matrix called PLI can be obtained.

##### 2.2.3.2 Coherence

The coherence (COH) measures the linear correlation of two signals in the frequency domain by calculating the cross spectral density and the self-spectral density of the two signals, and reflects the coupling intensity of the signals in the frequency domain. In EEG signal analysis, the coherence is widely used to study functional connections between different brain regions and can provide valuable information about the coordination of neural activity (Jun et al., 2021). Specifically, the COH (Equation 8) is defined as


(8) $$COH_{\mathrm{kl}}\left(f\right)=\frac{{\left|S_{\mathrm{kl}}\left(f\right)\right|}^{2}}{S_{kk}\left(f\right)S_{ll}\left(f\right)}$$

where $S_{\mathrm{kl}}\left(f\right)$ is the mutual spectral density of two channel signals $x_{k}\left(t\right)$ and $x_{l}\left(t\right)$ at frequency f . $S_{kk}\left(f\right)$ and $S_{ll}\left(f\right)$ are the self-spectral densities of $x_{k}\left(t\right)$ and $x_{l}\left(t\right)$ respectively.

The value range of the COH is from 0 to 1, and the larger the value is, the greater the correlation is between the two signals in the frequency domain. For K channel EEG signals, we can obtain a K×K COH functional connectivity matrix named COH by averaging $COH_{\mathrm{kl}}\left(f\right)$ at all frequencies, and set the value on the diagonal to zero by not considering the correlation between the two same channels. In this paper, K=19 .

##### 2.2.3.3 Functional connectivity matrix integrating PLI and COH

In EEG analysis, functional connectivity matrix is an important tool to reveal the interaction between different brain regions. It can reveal the dynamic characteristics of brain network by calculating the connection strength between the signals of the EEG channels. The PLI can effectively detect functional connections between brain regions, which is a very useful indicator. The COH provides information about frequency-domain connections between different brain regions, enabling the quantification of interactions between brain regions at specific frequencies. What is known is that building a functional connectivity matrix with metrics such as COH can reflect the amplitude synchronization relationship between the signals of the EEG channels, while it is strongly influenced by volume conduction artifacts (Murias et al., 2007). The PLI reduces the effect of volume conduction artifacts (Stam et al., 2007) and reflects the phase synchronization relationship between the signals of the EEG channels. Although the PLI and COH can provide functional connectivity information of different dimensions, it is difficult for a single indicator to fully reflect the complex interaction characteristics between brain regions. Therefore, in order to effectively circumvent the influence of volume conduction effect and capture the functional connectivity characteristics of brain networks more comprehensively, we fuse the PLI and COH to obtain a new feature (Equation 9) which is defined as


(9) $$P-COH_{\mathrm{kl}}=F\left(\mathrm{PL}I_{\mathrm{kl}}+COH_{\mathrm{kl}}\right)$$

where $\mathrm{PL}I_{\mathrm{kl}}$ and $COH_{\mathrm{kl}}$ are the $\left(k,l\right)th$ element in matrix PLI and COH respectively, and $F\left(\cdot \right)$ (Equation 10) is a mapping function that maps the value range of the fused feature from [0,2] to [0,1], which is defined as


(10) $$F\left(z\right)=\{\begin{array}{l} \frac{e^{z}-1}{2e-2},\left(0\leq z\leq 1\right)\\ 1-\frac{e^{2-z}-1}{2e-2},\left(1<z\leq 2\right) \end{array}$$

The purpose of the mapping function is to standardise the fused features while increasing their discriminative power. It enhances the discrimination between the fusion features of the patient and control participants and improves the effect of the new features by decreasing the values of smaller features and increasing the values of larger ones.

After mapping, the functional connectivity matrix P−COH based on the fusion features is obtained, which simultaneously reflects the phase synchronization and consistency of signal intensity in frequency domain between brain regions, and is the input of the GCN classification model. However, for the input of the GCN model, a binary matrix is required. It is important to set the appropriate threshold during the binarization process. In this study, we introduce a small-worldness as an index for threshold setting. We first set the threshold range between 0.3 and 0.7 with a step size of 0.01, then iterate all the thresholds and choose the value that maximizes the small-worldness as the threshold. Finally, we set the elements of the matrix P−COH greater than or equal to the threshold as 1 and the elements less than the threshold as 0.

By combining the PLI and COH to construct a functional connectivity matrix, we are able to capture more information about EEG signals simultaneously. This method can not only reduce the interference of volume conduction effect, but also effectively measure the connectivity characteristics between brain regions. With the nonlinear mapping, we can ensure that the resulting functional connectivity matrix has good normalization properties while retaining key information, which contributes to a more comprehensive assessment of the functional connectivity of brain networks and enables a more detailed understanding of brain neural communication.

### 2.3 GCN classification model

**Figure 4:**
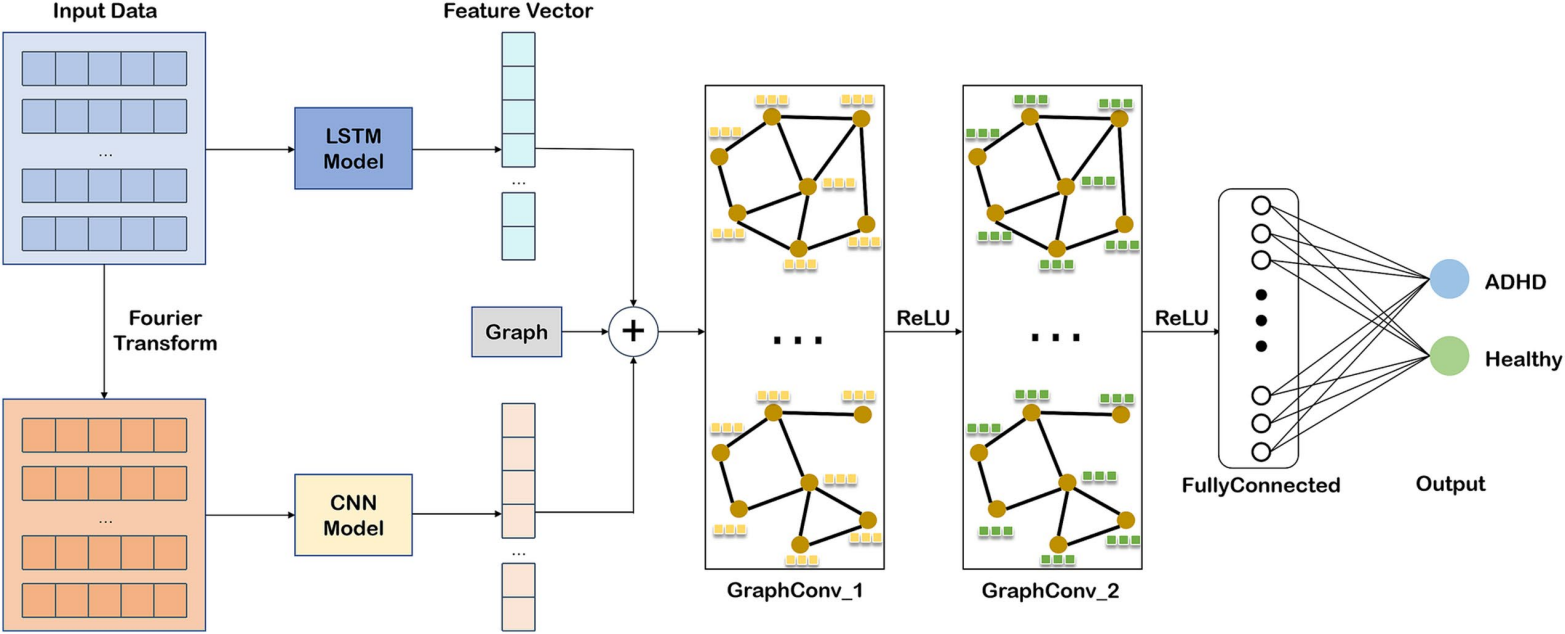
The schematic of the GCN classification model.

As a powerful deep learning framework, GCN is widely used in classification and recognition of graph structured data. In this study, we used a GCN classification model based on the mixed features and functional connectivity features to detect ADHD, which is shown in Figure 4.

The GCN model consists of graph convolution layers and pooling layers that play a role in reducing the dimensionality. The main purpose of the graph convolution layer is to aggregate the local neighborhood information of each node, so as to learn the relationship between the topology of the graph and node features. The graph convolution of each layer updates the node embeddings according to the characteristics of the node itself and its neighbors. The propagation rules (Equation 11) in GCN are defined by


(11) $$H^{\left(l+1\right)}=\mathrm{ReLU}\left({\tilde{D}}^{-\frac{1}{2}}\tilde{A}{\tilde{D}}^{-\frac{1}{2}}H^{\left(l\right)}W^{\left(l\right)}\right)$$

where, activation function $\mathrm{ReLU}\left(z\right)=\max\left(0,z\right)$ , $\tilde{A}$ is the adjacency matrix added with self-connection, $\tilde{A}=A+I$ , A is the adjacency matrix, I is the identity matrix, $\tilde{D}$ is the normalized form of the degree matrix of the matrix A , $H^{\left(l\right)}$ and $W^{\left(l\right)}$ are the node characteristic matrix and weight matrix of the layer l respectively.

In this study, we use the GCN model to detect ADHD including two graph convolution layers, each of which is followed by a pooling layer. The last pooling layer is followed by a global pooling layer and a fully connected layer with a dropout. The output is the classes for detecting the ADHD and healthy. Table 4 shows the parameter descriptions for the GCN model.

**Table 4:** Parameter descriptions for the GCN model.

Layer-model	GCN
Graph convolution	16
Pooling	16
Graph convolution	32
Pooling	32
Global pooling	32
Fully-connected	64
Dropout	50%
Output	2

In this paper, the functional connectivity matrix P−COH after binarizing is regarded as the adjacency matrix A of the graph convolution layer, where each node represents an EEG channel and the edge encodes the functional connections between the different channels. For 19 channels, the number of nodes of the graph convolution layer is 19. In order to enhance the expression ability of node features, we use the LSTM model and CNN model to obtain a 19×2 time domain feature matrix and a 19×2 frequency domain feature matrix, respectively. Finally, a 19×4 mixed feature matrix X is obtained, which is the input of the GCN model as the initial node characteristic matrix, that is $H^{\left(0\right)}=X$ .

### 2.4 Performance metrics

#### 2.4.1 Statistical analysis

The Mann–Whitney U test is a non-parametric statistical test used to assess whether there is a significant difference between two independent groups. It does not assume a normal distribution of the data and is appropriate for comparing ordinal or continuous data that do not meet the assumptions of parametric tests. As we know, the *p*-value obtained from the test is used to determine the statistical significance of the difference in feature distributions. A *p*-value less than 0.05 suggests that the difference between the two groups is statistically significant, indicating substantial dissimilarity in the features between the patient and normal groups. As mentioned above, we use fusion features as a new marker to construct the functional connectivity matrix in this study, and we use the Mann–Whitney U test method to verify its validity.

#### 2.4.2 Evaluation index

In order to better evaluate the classification performance of the proposed method, we chose accuracy (Equation 12), recall (Equation 13) (also known as sensitivity), and precision (Equation 14) (also known as specificity) as the evaluation metrics in this paper. These three indicators are defined, respectively, as


(12) $$\mathrm{accuracy}=\frac{TP+TN}{TP+TN+FP+FN}$$

(13) $$\mathrm{recall}=\frac{TP}{TP+FN}$$

(14) $$\mathrm{precision}=\frac{TP}{TP+FP}$$

where TP , TN , FP and FN are true positive, true negative, false positive and false negative, respectively.

## 3 Experimental results and discussion

### 3.1 Experimental results

#### 3.1.1 Mann–Whitney U test results

We conduct Mann–Whitney U test on the functional connectivity matrix constructed based on Dataset 1 and Dataset 2, and a small *p*-value indicates a large difference in features. Table 5 shows the *p*-values of two datasets. The results show that *p*-values of both datasets are far less than 0.05, which means that the proposed method has good effectiveness on both datasets, and the *p*-value of Dataset 2 is far less than the *p*-value Dataset 1, indicating that the proposed method has more obvious effect on Dataset 2. We notice that the difference in *p*-values between the two datasets is very obvious, indicating that although our method achieves good results on both datasets with a small number of participants, the effect on each group of data is not completely consistent. The key to solving this problem may be to conduct experiments with large-scale datasets in the future.

**Table 5:** The *p*-values of two datasets.

Measure	Dataset 1	Dataset 2
*p*-value	2.53e-65	1.87e-189

#### 3.1.2 Classification results of two datasets

On both datasets, we use five-fold cross-validation and conduct Bootstrap analysis on the model. The sample size of Bootstrap is set to 1,000 times and the confidence interval is set to 95%. During GCN network training, the number of epochs is 100, batch size is 64, Adam optimizer is used with an initial learning rate of 1e-3 and a binary cross-entropy loss function is used as the loss function.

**Figure 5:**
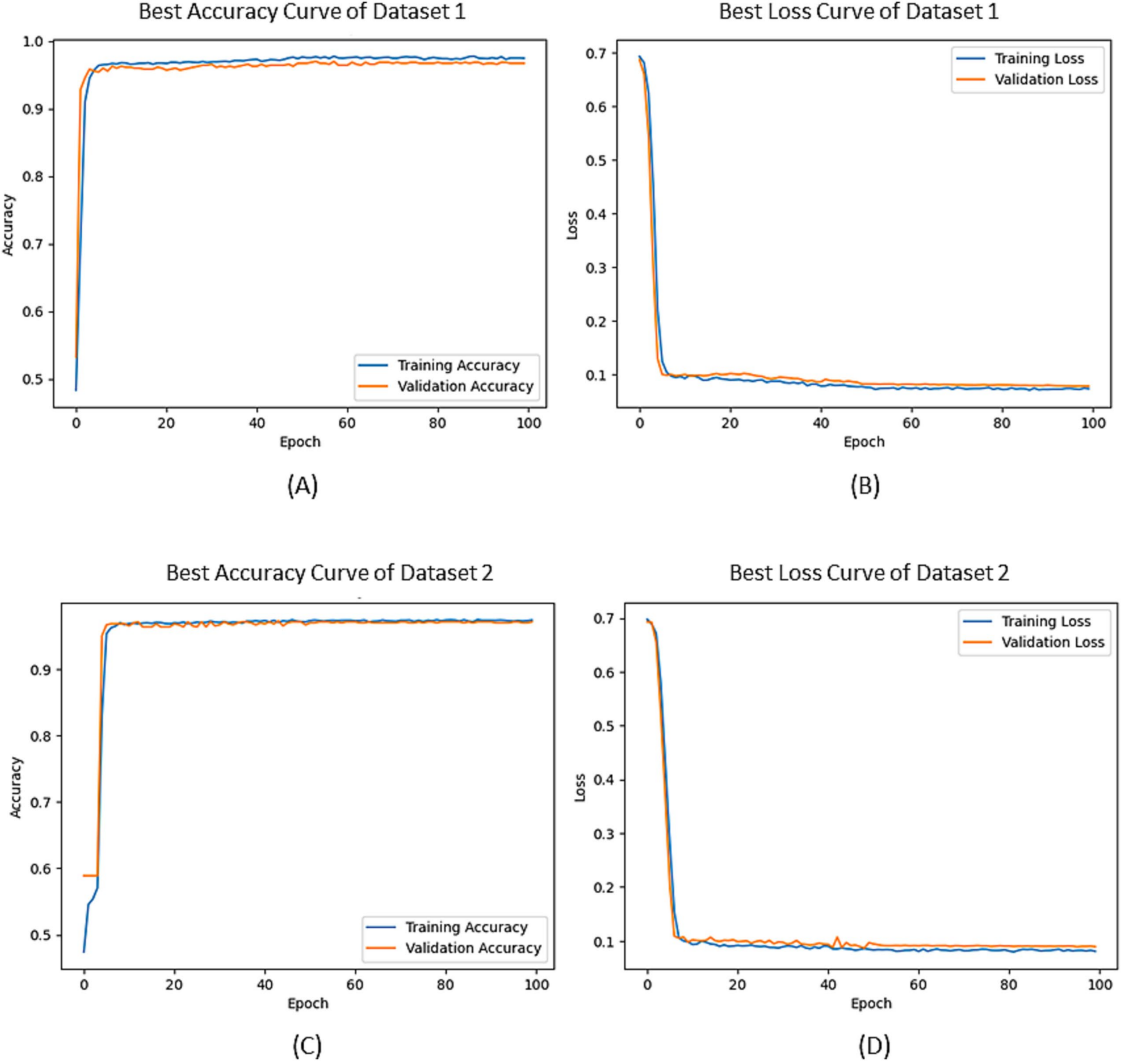
The accuracy and loss curves: **(A)** Classification accuracy curves of Dataset 1; **(B)** loss curves of Dataset 1; **(C)** Classification accuracy curves of Dataset 2; **(D)** loss curves of Dataset 2.

In Figure 5, the optimal accuracy and loss function curves of both datasets are given after the five-fold cross-validation. From the figure, we can see that the accuracy curves on the training set and the verification set do not have a large deviation, which indicates that the degree of overfitting of our model is small. The results of Bootstrap analysis of the model are shown in Table 6. The results show that the proposed method performs well on both datasets, resulting in the highest accuracy rates of 97.83 and 97.45%, the highest recall scores 97.84 and 97.36%, and the highest precision scores 98.53 and 98.58%, respectively. Moreover the scores of the five folds from the confidence interval of 95% are not significantly different, indicating that the model is robust.

**Table 6:** Bootstrap analysis results of Dataset 1 and Dataset 2.

Dataset 1	Accuracy (%)	Recall (%)	Precision (%)
Fold 1	96.54 (95.11, 97.84)	96.36 (94.38, 98.03)	96.73 (94.80, 98.46)
Fold 2	97.56 (96.41, 98.71)	96.74 (95.00, 98.37)	98.47 (96.99, 99.69)
Fold 3	96.69 (95.25, 97.99)	95.69 (93.33, 97.89)	97.57 (95.89, 98.96)
Fold 4	**97.83 (96.69, 98.85)**	97.15 (95.24, 98.76)	**98.53 (97.08, 99.71)**
Fold 5	97.82 (96.55, 98.85)	**97.84 (96.17, 99.20)**	97.82 (96.05, 99.12)
Average	97.29 (96.00, 98.45)	96.76 (94.82, 98.45)	97.82 (96.16, 99.19)

Dataset 2	Accuracy (%)	Recall (%)	Precision (%)
Fold 1	97.43 (96.24, 98.65)	**97.36 (95.76, 98.76)**	97.53 (95.64, 99.30)
Fold 2	95.22 (93.52, 96.84)	94.72 (92.33, 96.95)	95.79 (93.31, 98.01)
Fold 3	96.10 (94.43, 97.59)	95.63 (93.39, 97.64)	96.61 (94.46, 98.48)
Fold 4	97.17 (95.78, 98.34)	96.98 (95.27, 98.48)	97.45 (95.32, 99.20)
Fold 5	**97.45 (96.24, 98.65)**	96.64 (94.74, 98.38)	**98.58 (97.08, 99.69)**
Average	96.67 (95.24, 98.01)	96.27 (94.30, 98.04)	97.19 (95.16, 98.94)

The bold values represent the highest metrics of the 5 folds.

#### 3.1.3 Comparisons of proposed method against other models

In this section, we compare our model with other traditional machine learning (ML) algorithms such as XGBoost (Chen and Guestrin, 2016), LightGBM (Ke et al., 2017), AdaBoost (Cao et al., 2013) and random forest (Breiman, 2001) to show the superiority of our model with five-fold cross-validation. We use the LSTM and CNN models mentioned above to extract the time domain and frequency domain features as the inputs of these four ML algorithms. The comparison results on Dataset 1 and Dataset 2 are shown in Tables 7, 8 respectively. The results show that our designed model performs much better than the other models, achieving the average accuracy rates 97.29 and 96.67%, the average recall scores 96.76 and 96.27%, and the average precision scores 97.82 and 97.19% on Dataset1 and Dateset2, respectively, using five-fold cross-validation. Among the traditional ML algorithms, XGBoost algorithm has slightly better performance than the other algorithms, resulting in the average accuracy rate 83.35%, the average recall scores 82.43% and the average precision score 83.56% on Dataset 1. While random forest algorithm obtains second best performance with the average accuracy 75.53%, the average recall 77.65% and the average precision 76.85% on Dataset 2.

**Table 7:** Comparisons of the proposed method with other models on Dataset 1.

Metric	XGBoost	LightGBM	AdaBoost	Random forest	Proposed
Accuracy (%)	83.35	80.81	78.45	81.23	97.29
Recall (%)	82.43	78.35	76.21	80.15	96.76
Precision (%)	83.56	78.93	77.53	81.14	97.82

**Table 8:** Comparisons of the proposed method with other models on Dataset 2.

Metric	XGBoost	LightGBM	AdaBoost	Random Forest	Proposed
Accuracy (%)	74.69	73.89	72.56	75.53	96.67
Recall (%)	73.83	70.78	73.35	77.65	96.27
Precision (%)	75.43	72.81	72.93	76.85	97.19

#### 3.1.4 Ablation experiments

In order to explore the influence of different feature components on the performance of the model proposed in this study, we set up ablation experiments with five-fold cross-validation. We quantify the impact of these components on the model performance by gradually removing or replacing the key components of the model, such as the time domain features, frequency domain features, and GCN layers. Specifically, we set up three experiments, including only using frequency domain or time domain features as the inputs of the model, and reducing one GCN layer, respectively. All experiments are performed on the same training set and verification set to ensure a fair comparison. The experimental results are shown in Table 9.

**Table 9:** Results of ablation experiments.

Dataset 1	Accuracy (%)	Recall (%)	Precision (%)
Baseline model (full model)	97.29	96.76	97.82
Without time domain features	95.65	96.73	95.21
Without frequency domain features	96.23	96.53	97.15
Reduced a GCN layer	81.53	82.87	81.34

Dataset 2	Accuracy (%)	Recall (%)	Precision (%)
Baseline model (full model)	96.67	96.27	97.19
Without time domain features	95.12	94.43	95.86
Without frequency domain features	96.21	95.12	96.65
Reduced a GCN layer	79.11	78.21	81.52

The results show that the performances of the three experimental models have decreased. Without the time domain features, the accuracy rates have decreased by 1.64 and 1.55%, the recall scores have decreased by 0.03 and 1.84%, and the precision scores have decreased 2.61 and 1.33% on Dataset 1 and Dataset 2, respectively. Without the frequency domain features, the accuracy rates have decreased by 1.06 and 0.46%, the recall scores have decreased by 0.23 and 1.15%, and the precision scores have decreased 0.67 and 0.54% on Dataset 1 and Dataset 2, respectively. Reducing one GCN layer, the accuracy rates have decreased by 15.76 and 17.56%, the recall scores have decreased by 13.89 and 18.06%, and the precision scores have decreased 16.48 and 15.67% on Dataset 1 and Dataset 2, respectively. These results demonstrate that integrated multi-domain features can improve the performance of the proposed model. We can see that although the performances of the experimental models without time domain features or frequency domain features do not decrease much, they fail to reach the performance with both time domain and frequency domain features, which indicates that the features of a single scale are limited for the model. In addition, reducing one GCN layer model has the greatest performance degradation, which indicates that the complexity of the current GCN structure is reasonable.

## 4 Discussion

### 4.1 Analysis of brain connectivity patterns

**Figure 6:**
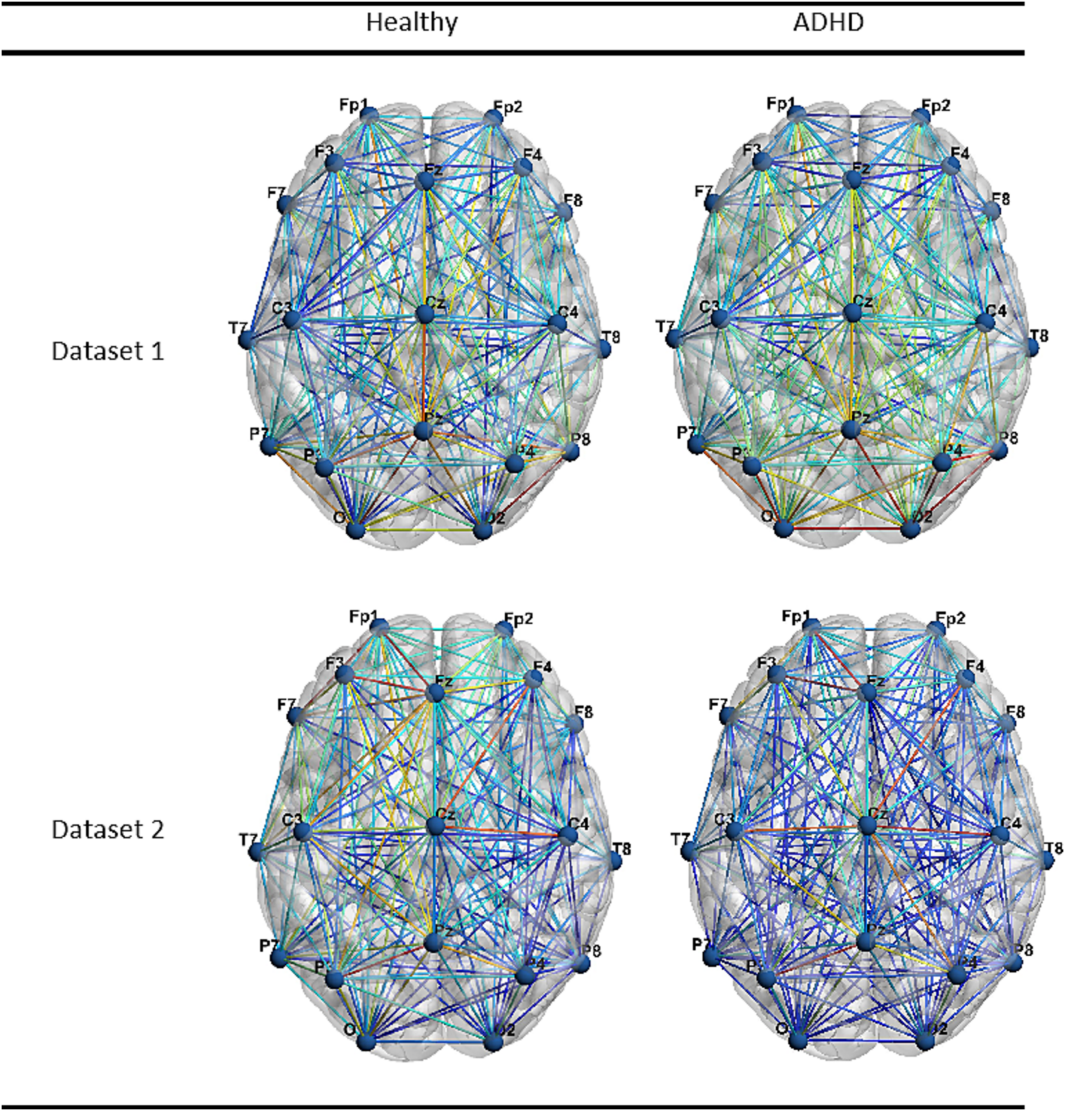
The functional brain connectivity maps of two datasets.

In this section, we use BrainNet Viewer for visualizing and analyzing brain connections. We average the functional connectivity matrices of all the samples, and a higher value of the matrix elements represents a tighter connection between two channels. This method not only visualizes the general location of each electrode on the brain, but also shows the connections between different brain regions more clearly, which is benefit for clinical interpretability. Figure 6 shows the functional brain connectivity maps of children with ADHD and children in the control group in Dataset 1 and Dataset 2. The red line represents a larger value, while the blue line represents a smaller value. It is worth noting that Dataset 1 is the resting state EEG data and Dataset 2 is the task state EEG data, which can provide a richer reference for our research.

ADHD children in Dataset 1 have more warm color connections in the functional brain connectivity maps, which means that the overall functional brain connectivity of ADHD children in the resting state is stronger, while ADHD children in Dataset 2 have more cold color connections in the functional brain connectivity maps, indicating that the overall functional brain connectivity of ADHD children is significantly lower during the task. This phenomenon may explain the inability of ADHD children to adjust to task demands. The study on dynamic brain connectivity in ADHD based on fNIRS (Sutoko et al., 2020) used a probabilistic model to reveal the connectivity states, and concluded that the probability of brain connectivity in ADHD children in two major task-related states decreased, while the probability of occurrence in two task-independent states increased. Our results coincide with the above conclusions.

What we do know is that different regions of the brain have different functions. The frontal lobe is involved in a wide range of cognitive functions such as attention. The occipital lobe is responsible for receiving and integrating visual information. The temporal lobe is responsible for processing auditory and verbal information as well as having advanced visual abilities, and the parietal lobe is capable of processing all types of sensory information. From Figure 6, we can observe that ADHD children in both Dataset 1 and Dataset 2 have low frontal connectivity, and the abnormality of this region has a significant impact on the cognitive function of patients (Michalka et al., 2015), which may be one of the significant signs of brain connectivity in ADHD patients. ADHD children in Dataset 1 have relatively strong long-range frontal–parietal and frontal-occipital connections, and the frontal–parietal connection is considered to be a critical attention network (Corbetta et al., 2008). Furthermore, the connectivity of the frontal and occipital lobes can effectively regulate the processing of cognitive information (Siegel et al., 2008). In the resting state, the connectivity of these two long-range connections is abnormal, which further indicates that ADHD patients cannot adjust the functional regions of the brain according to demand. The occipito-temporal pathway contributes to the redirection of attention to obvious, behavior-related external stimuli, while the fronto-parietal network is responsible for the goal-oriented execution of tasks (Faraone et al., 2021). The occipito-temporal and fronto-parietal connectivity of ADHD children in Dataset 2 is underactivated. This may also be one of the abnormal characteristics of brain connectivity in ADHD patients under task state.

Our study reveals a link between connectivity between different brain regions and attention deficit disorder, and more research in this area may lead to a deeper understanding of the underlying causes of attention deficit disorder.

#### 4.1.1 Limits and future directions

Although the proposed method based on the multi-domain features in this paper has made good progress in the detection of ADHD, most of the current automated ADHD detection technologies still face some significant challenges. What we all know is that the robustness of deep learning depends on the support of large-scale datasets. However, building a large medical dataset is a difficult task. Therefore, the results of this study are affected by the limitation of the size of dataset. Using a broader dataset rather than a limited dataset can enhance the efficiency of the proposed method and provide more accurate results. In addition, the EEG signal has the advantages of ultra-high temporal resolution, portable and cheap equipment, but it also has the problem of low spatial resolution, and it is weak to rely solely on EEG signals to mine the potential pathological information of ADHD. Therefore, we will try to combine other medical imaging methods, such as fMRI, to construct a multi-modal dataset in future. This may lead to more help for ADHD detection. By in-depth analysis of high-quality features in the different modal data, we expect to be able to more fully reveal the details of the abnormal neural mechanisms of ADHD.

## 5 Conclusion

In this study, we propose a new method for ADHD detection using EEG in which the time domain and frequency domain features are extracted by a LSTM model and a CNN model respectively, a functional connectivity matrix is constructed by a new indicator through integrating the PLI and COH, and then a GCN model is used to distinguish the ADHD and healthy children. On the Dataset 1 collected by ourselves and the public Dataset 2, the new functional connectivity is verified that it can be used to recognize ADHD by Mann–Whitney U test. Moreover, the proposed method shows excellent classification performance in terms of the accuracy, recall, and precision by Bootstrap analysis, and has better classification results than other methods, such as XGBoost, LightGBM, AdaBoost and random forest. The ablation experiments show that the fused multi-domain features can improve the performance of the proposed method. The proposed method can shorten the process of traditional diagnosis and help neurologists make more accurate diagnoses. In addition, few previous studies have examined brain connectivity in children with ADHD using both resting state EEG data and task state EEG data, but this is very important. We find that in the resting state, the brain connectivity of children with ADHD increased overall, while in the task state, their brain connectivity decreased overall. This conclusion reveals the differences in brain networks of children with ADHD in different states. Certainly, there are still some limitations in our study at this stage, and in the future, our study will combine other medical imaging techniques to help us understand ADHD more comprehensively, and strive to make new contributions to the study and diagnosis of ADHD.

## Data Availability

Data were kept anonymized and used only for analysis purposes to ensure participant confidentiality. Ethical approval did not include publication of Dataset 1, therefore it cannot be shared.
