# Corrigendum: ADHD detection from EEG signals using GCN based on multi-domain features

**DOI:** 10.3389/fnins.2025.1621212

**Published:** 2025-05-13

**Authors:** Ling Li, Xueyang Guo, Zihan Yang, Yanping Zhao, Xu Liu, Junxian Yang, Yanyan Chen, Xinxian Peng, Lina Han

**Affiliations:** ^1^College of Communication Engineering, Jilin University, Changchun, China; ^2^College of Geo-exploration Science and Technology of Jilin University, Changchun, China; ^3^Changchun Sixth Hospital of China, Changchun, China

**Keywords:** ADHD, EEG, brain functional connectivity, multi-domain features, GCN

In the published article, there was an error in the Funding statement. The grant number for the Jilin Province Health and Technology Capacity Enhancement Project (CN) was erroneously displayed as “222Lc132”. The correct Funding statement appears below.

